# Surgical resection of an intradural extramedullary spinal tumor

**DOI:** 10.3171/2023.7.FOCVID2351

**Published:** 2023-10-01

**Authors:** Joseph Yunga Tigre, Adam Levy, Eva M. Wu, James Boddu, Vignessh Kumar, Allan D. Levi, S. Shelby Burks

**Affiliations:** 1 Department of Neurosurgery and; 2 The Miami Project to Cure Paralysis, University of Miami Miller School of Medicine, Miami, Florida

**Keywords:** intradural spinal tumor, spinal meningioma, surgical resection

## Abstract

Large ventrally located spinal meningiomas are typically resected via a posterolateral or lateral approach. Optimal outcomes are associated with good preoperative functional status (i.e., modified McCormick grade < 4), while recurrence rates may be predicted by degree and quality of resection (i.e., low Simpson grade). This video describes the operative techniques for resection of a large ventral C2 intradural extramedullary meningioma in a 71-year-old male presenting with hemibody sensory loss and abnormal gait. A paramedian approach was performed, allowing for adequate exposure and gross-total resection. The patient was discharged on postoperative day 2 and showed near-complete resolution of sensory deficits.

## Transcript

We’re presenting a case of a surgical resection of an intradural, extramedullary spinal tumor.

### 0:27 Clinical Presentation.

The patient is a 71-year-old right-handed male who presented for evaluation of left arm numbness and paresthesia. Left upper-extremity paresthesia began 2 years ago and radiated into the shoulder and hand. He underwent left carpal tunnel release without significant benefit, and symptoms progressed to ultimately include difficulty ambulating over the last several months. His past medical and surgical history are included below.

On physical exam, the patient demonstrated normal mentation and an unsteady gait. He had difficulty with heel and toe walking as well as tandem walking. On motor examination, he generally had full strength except for left hip flexor and left-hand grip, both of which demonstrated subtle weakness. He had diffusely elevated reflexes throughout with positive Hoffmann’s signs bilaterally. On sensory examination, he had reduced sensation to light touch in the left upper extremity in a nondermatomal distribution.

### 1:15 Preoperative Imaging.

The MRI scan is now presented with the sagittal image T2-weighted seen here. Large ventral lesion based on the dura at approximately the level of C2 causing severe compression of the spinal cord. This can be further illustrated on the axial image T2-weighted. As we scroll, we see a left-sided dural-based lesion, ventrally based, causing severe compression of the spinal cord.

Here we see an axial and sagittal T1 with gadolinium image demonstrating homogeneous enhancement of this lesion.

### 1:52 Considerations, Risks, and Benefits.

Diagnostic considerations are presented here with the C1–2 intradural extramedullary lesion, likely a meningioma. Our recommendation is a C1–3 laminectomy with removal of this intradural extramedullary tumor.

The rationale for this procedure is progressive symptomatology and spinal cord compression.

The risks are not limited to but include bleeding, infection, spinal cord injury, spinal fluid leak, development of postoperative cervical kyphosis and neck pain, as well as a need for additional revision surgery. The benefits of surgery include decompression of the spinal cord, relief of symptoms, and tumor control with establishment of diagnosis.

Alternative treatments in this case are not suggested, and surgery is strongly recommended given the degree of spinal cord compression, but in cases of patients unable to tolerate surgery, continued surveillance and symptoms control could be considered.

### 2:43 Operative Preparation.

For setup of this case, we’ll use a prone position with pinning of the head using a Mayfield head holder and padding of all pressure points, including bilateral ulnar nerves and peroneal nerves. The equipment needed includes intraoperative neurological monitoring with SSEP and MEPs, as well as free-run EMGs, neuromonitoring signals were checked every 30 minutes through the duration of the case, intraoperative sterile ultrasound, operating microscope, and an ultrasonic aspiration device. The key surgical steps: first, meticulous muscle dissection, which is especially important in the posterior cervical region, followed by a C1–3 laminectomy with exposure of the dura. Ultrasound is then used to confirm the cranial and caudal exposure of the tumor. Following this, a microscope is brought into the field, and then a paramedian durotomy is created. The tumor resection is then carried out using a combination of instruments, including the ultrasonic aspirator. Following removal of the tumor and bipolaring of the dural base, watertight dural closure is performed with multilayered closure of the muscle and fascia.

### 3:44 C1–3 Laminectomy.

Laminectomy from C1 to C3 was conducted in the usual fashion, with a midline approach, and the joints were preserved at each of these levels.

### 3:53 Intraoperative Ultrasound.

A portion of the intraoperative ultrasound is depicted here with an axial view where the spinal cord can be seen severely displaced to the right by this large ventral tumor. Again, the ultrasound was utilized to confirm that we were cranial and caudal to the tumor with our exposure and our laminectomy.

### 4:12 Dural Incision.

Again, the surgery begins with a paramedian dural opening. Centered off to the left side, which can be seen here, utilizing a combination of instruments, including suctions, the grooved director, as well as forceps, including jeweler and microbayoneted forceps.

Once the dura is opened, we’re able to tack this up using small sutures. In this case, Prolene sutures were utilized, 5-0 Prolene. These would be then tacked up to the paraspinal muscles. Here you can see the arachnoid and also the tumor. As the arachnoid is opened, the tumor is better visualized. Here you see us retracting the spinal cord slightly toward the right and establishing a plane between the spinal cord and tumor. As this plane is better established, we were able to debulk the tumor. We did identify the spinal accessory nerve, which will be shown shortly, as is seen here, as well as the C2 nerve root. The spinal accessory nerve, in this case, will be flipped medially and laterally as the tumor is resected.

### 5:17 Debulking of Tumor.

Through debulking the tumor, we again use a combination of instruments, with the main instruments being the bipolar, irrigating bipolar in this case, as well as the ultrasonic aspirator, which will be seen shortly. Rhoton instruments allow us to retract the spinal cord very gently, and this window is maintained throughout the case. The microscissor also is utilized to section small bands within the tumor. Using the microtip on the ultrasonic aspirator was important in this case as our window in between the spinal cord and edge of the dura was relatively small.

### 5:55 Resection of Tumor.

Here as we’ve resected part of the tumor, we’re attempting to mobilize it and drag a portion of the tumor into the small window that we have. In this case, we’ve removed the most cranial portion of the tumor, and we’re now working toward the caudal piece of the tumor, which lies under the spinal cord and ventral to spinal cord. Maintaining the plane between the spinal cord and tumor is very important. Here you can also see where the tumor originates from the ventral dura. The ventral dura is visualized there, along with intradural veins dorsal to the spinal cord. Throughout the tumor resection, MEPs are utilized at 30-minute intervals to confirm that there’s been no changes in spinal cord signals. Now the tumor is being reflected away from the spinal cord, and our corridor is maintained slightly widened at this part as the tumor is debulked. The tumor is now being resected with the ultrasonic aspirator and further dragged into the field. The spinal accessory nerve is visualized on the lower portion of the screen and is being kept safe with the suction. The last remaining portion of the tumor is swept into the field, and its dural base identified. Microcurettes can be utilized to scrape and detach the tumor from the intradural veins as well as its dural attachment. We then bipolar any remaining tumor on the dura, as well as the dural base of the tumor.

This helps prevent tumor recurrence and enables our gross-total resection. Continuous irrigation is utilized, as well as Gelfoam patties in this case, to coagulate any bleeding. Once the bleeding has subsided, we then close the dura in a watertight fashion again, using Prolene 5-0 sutures, with intermittent locking sutures to maintain tension on the dural edges. Watertight closure was achieved in this case, and after fibrin glue was then sprayed over the dural repair site.

### 8:21 Postoperative Care.

Following dural closure, the fascia was closed in a watertight fashion with a Hemovac drain placed in the epidural space. Skin and subcutaneous tissue was subsequently closed with a Monocryl suture under the skin, and Dermabond glue used over the skin edges. After closure we evaluated the wound and determined there was no instability generated from the laminectomy, as it was a typical midline-style laminectomy without any joint removal.

### 8:48 Discussion of Pathology.

Primary spinal tumors comprise 2%–4% of all CNS tumors.^1^ These can be classified based on their anatomical locations.^2^ Meningiomas and schwannoma are the most common intradural extramedullary tumors to occur.^3^ Regarding spinal meningiomas, symptoms are often nonspecific, with radicular symptoms and back pain often occurring.^4^ Slowly progressive neurological deficits can happen, as was the case with our patient. Management includes surgical treatment, which is favored, and complete resection results in excellent functional outcomes and low recurrence rates.^5,6^

### 9:20 Postoperative Course.

The postoperative course in our patient. We had no perioperative complications; this patient was discharged on POD 2 with near-complete resolution of neurological deficits. Physical exam at 2 weeks demonstrated an improvement in his examination as well as stable weakness in the left hand and left intrinsics. The skin was clean, dry, and intact, and the wound was healing well. The final pathology was meningioma WHO grade 1. Histology showed no features of atypia and a Ki-67 index of 1%.

Postoperative MRI is illustrated here with the sagittal T2 image showing the laminectomy defect and no evidence of residual tumor. We see the axial image T2 weighted. Again, there’s a small area of myelomalacia where the tumor was causing severe compression. We’ll also now see the sagittal and axial postgadolinium images showing no residual gadolinium enhancement. We do see a small amount of enhancement in the paraspinal muscles, as well as a small pseudomeningocele, which is common after intradural surgeries. The patient reported no issues, and healing was routine.

### 10:26 Conclusions.

In conclusion, surgical removal of intradural tumors allows for rapid decompression and stands as an effective means of symptom relief in these types of patients. Adequate exposure is essential in achieving gross-total resection with cranial and caudal exposure of the tumor. Use of intraoperative imaging, such as ultrasound, can assist with this as well as other intraoperative adjuncts, as are illustrated in this case. Thank you.

## Disclosures

Dr. Levi receives a teaching honorarium from the American Association of Neurological Surgeons and grant support from the Department of Defense and the National Institutes of Health (NIH-NINDS).

## Author Contributions

Primary surgeon: Burks. Assistant surgeon: Boddu, Kumar, Levi. Editing and drafting the video and abstract: Burks, Yunga Tigre, Levy, Wu, Boddu, Kumar, Levi. Critically revising the work: Burks, Levy, Wu, Boddu, Kumar, Levi. Reviewed submitted version of the work: all authors. Supervision: Burks, Levi.

## Supplemental Information

### Patient Informed Consent

The necessary patient informed consent was obtained in this study.
